# Non-Covalent Dimer as Donor Chromophore for Constructing Artificial Light-Harvesting System in Water

**DOI:** 10.3390/molecules27248876

**Published:** 2022-12-14

**Authors:** Liangliang Zhang, Hongwei Qian, Zhiying Wu, Qiaona Zhang, Shengke Li, Ming Cheng, Tangxin Xiao

**Affiliations:** 1 Jiangsu Key Laboratory of Advanced Catalytic Materials and Technology, School of Petrochemical Engineering, Changzhou University, Changzhou 213164, China; 2 State Key Laboratory of Quality Research in Chinese Medicine, Institute of Chinese Medical Sciences, University of Macau, Macau 999078, China; 3 Jiangsu Key Laboratory of Advanced Organic Materials, School of Chemistry and Chemical Engineering, Nanjing University, 163 Xianlin Avenue, Nanjing 210023, China

**Keywords:** aggregation-induced emission, quadruple hydrogen bonding, non-covalent dimer, light-harvesting system, nanoparticles

## Abstract

Dynamic emissive materials in aqueous media have received much attention owing to their ease of preparation, tunable luminescence and environmental friendliness. However, hydrophobic fluorophores usually suffer from aggregation-caused quenching in water. In this work, we constructed an artificial light-harvesting system by using a non-covalent aggregation-induced emission dimer as antenna and energy donor. The dimer is quadruple hydrogen bonded from a ureidopyrimidinone derivative (**M**) containing a tetraphenylethylene group. The dispersed nano-assemblies based on the dimer in aqueous media were fabricated with the help of surfactant. By loading a hydrophobic acceptor molecule **DBT** into the nano-assemblies, man-made light-harvesting nanoparticles were fabricated, showing considerable energy transfer efficiency and a relatively high antenna effect. Additionally, the fluorescence color of the system can be gradually tuned by varying the content of the acceptors. This study provides a general way for the construction of an aqueous light-harvesting system based on a supramolecular dimer, which is important for potential application in luminescent materials.

## 1. Introduction

With the energy crisis becoming more and more serious around the world, the exploitation and development of solar energy has received considerable attention. In this context, a series of artificial light-harvesting systems (LHSs) were constructed by mimicking natural photosynthesis for various applications, such as bioimaging, sensing, photocatalysis and manufacturing luminescent materials [1,2,3,4,5,6]. In most natural photosynthetic organisms, for example, chlorosome, the rigid protein scaffolds play a critical role for binding pigments and controlling their excited energy transfer. Therefore, in order to mimic nature and avoid aggregation-caused quenching of fluorophores, a variety of man-made scaffolds, such as host–guest complexes [7,8,9,10,11,12], coordination macrocycles [13,14,15,16], supramolecular polymers [17,18,19,20,21,22] and biomacromolecules [23,24], were created or used to accommodate chromophores in artificial LHSs. However, the design and synthesis of an elegant scaffold is difficult. To address this challenge, the employment of aggregation-induced emission (AIE) [25,26,27,28,29] greatly simplifies the fabrication steps of special scaffolds [30,31,32,33,34]. It is noteworthy that organic AIE molecules dispersed as random nanoparticles (NPs) in an aqueous media instead of in a specific scaffold show strong fluorescence, which might serve as a nano-platform for constructing LHSs.

Tetraphenylethylene (TPE) is a typical AIE group. Our previous reports show that NPs formed by the TPE monomer show poor light harvesting capability [35,36]. To solve this problem, the covalently bonded TPE dimers are used to form water-dispersed NPs and act as a light-harvesting antenna. However, the synthesis of TPE dimer is tedious and time-consuming. Therefore, we wondered whether non-covalent dimers could be an alternative way to fabricate LHS. Quadruple hydrogen bonds based on the ureidopyrimidinone motif show great potential in supramolecular chemistry owing to their strong binding ability and self-complementary behavior [37,38]. In our previous works, quadruple hydrogen bonds were employed for the construction of supramolecular polymers [39,40] and other interesting supramolecular entities [41,42]. In this study, we constructed an artificial LHS by utilizing quadruple hydrogen bonded AIE dimers as a light-harvesting antenna and excitation energy donor.

**Scheme 1:**
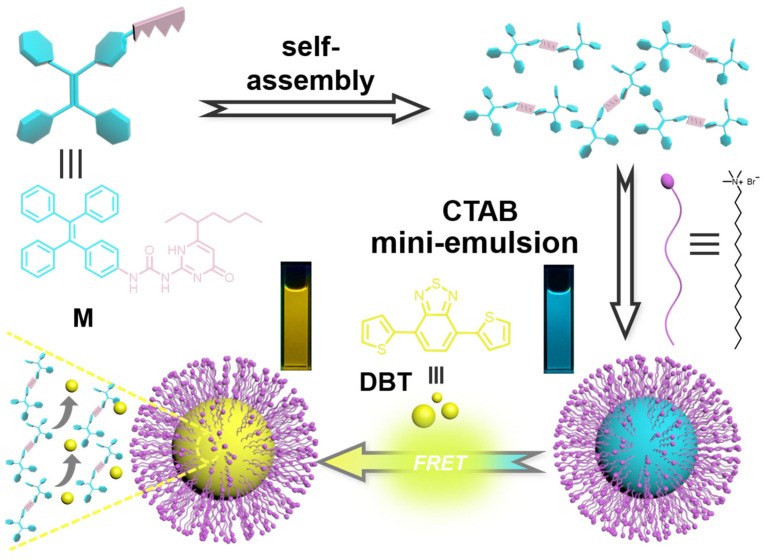
Schematic representation of dimerization of **M**, self-assembly of the NPs and the energy-transfer process with **DBT**, as well as the chemical structures of **M**, **CTAB** and **DBT**.

The monomer is a TPE group derived ureidopyrimidinone (UPy) compound (**M**), which serves as the energy donor (Scheme 1). Notably, the TPE group provides **M** with the AIE property, while the UPy moiety endows **M** with dimerization capability through quadruple hydrogen bonding. Therefore, **M** can form quadruple hydrogen bonded supramolecular dimers in chloroform. Subsequently, the non-covalent dimers were further assembled into NPs in the aqueous media by the mini-emulsion approach [43,44], which is assisted by the amphiphile cetyltrimethyl ammonium bromide (**CTAB**). By loading a commercially available dye **DBT** (4,7-di(2-thienyl)-benzo [2,1,3]thiadiazole) into the NPs to serve as the acceptor, a supramolecular LHS in water was successfully constructed. Owing to the Förster resonance energy-transfer (FRET) process from **M** to **DBT**, the NPs display considerable energy transfer efficiency and tunable emission property. Herein, the **M** molecule plays triple roles including supramolecular dimerization, light-harvesting antenna and excitation energy donor. As a result, the **M**-**DBT** dual-chromophore system was successfully used to construct an efficient LHS in water. Such a system will be an interesting prototype nanoplatform for light harvesting and may have potential applications in the field of organic luminescence and biological imaging materials.

## 2. Results and Discussion

**Figure 1:**
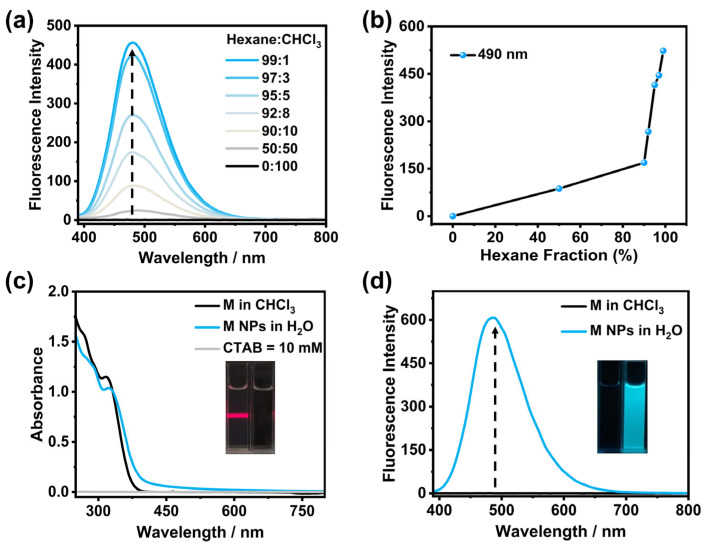
(**a**) Fluorescence spectra of **M** in mixed hexane/CHCl_3_ solvents at different vol% upon excitation at 330 nm. (**b**) Fluorescence intensity of **M** at 490 nm in the presence of different factions of hexane in mixed solution. (**c**) UV-Vis spectra of **M** in CHCl_3_ and the prepared **M** NPs in water, inset: Tyndall effect of **M** NPs in water (left) and **M** in CHCl_3_ (right). (**d**) Fluorescence spectra of **M** in CHCl_3_ and in water, inset: fluorescence images of **M** in pure CHCl_3_ (left) and water (right). [**M**] = 5 × 10^−5^ M.

The synthesis of **M** is straight forward, and it was fully characterized by ^1^H NMR, ^13^C NMR and high-resolution ESI-MS (Figures S1–S3). On account of the UPy unit, the **M** monomer can undergo dimerization through quadruple hydrogen bonding interaction, which was confirmed by the ^1^H NMR experiment. As can be seen from Figure S2, N–*H* protons of UPy appear in the very downfield region (between 12.0 and 14.0 ppm) in CDCl_3_, indicating that strong hydrogen bonds are formed between monomers through UPy units. Subsequently, the AIE behavior of **M** was further investigated in mixed CHCl_3_/hexane solvent. As revealed by the fluorescence spectra (Figure 1a), **M** shows a very weak fluorescence in pure CHCl_3_, indicating that **M** is molecularly dissolved in pure CHCl_3_. However, a slight increase in emission intensity was recorded when the hexane fraction (vol%) reached 50%. As the hexane fraction continued to increase to 99%, a significant emission enhancement was observed. The change in the fluorescence intensity of **M** at 490 nm upon the hexane fraction was plotted in Figure 1b. The emission enhancement occurs mainly because the rotation of TPE groups in **M** is limited and the non-radiation inactivation is reduced, which indicates that **M** is a typical AIE molecule.

The good AIE property of **M** encourages us to prepare fluorescent NPs of **M** in water via the mini-emulsion method. The formation of nanoaggregates was first confirmed by an obvious Tyndall effect (Figure 1c, inset). In contrast, the solution of **M** in CHCl_3_ exhibited no Tyndall effect due to molecular dissolution of **M**. Moreover, the UV-Vis absorption peak of aqueous solution of the NPs was similar with the CHCl_3_ solution of **M** at approximately 330 nm, indicating the **M** dimers were successfully dispersed in **CTAB** micelles. Notably, the absorbance spectrum of the NPs exhibited a high energy tail at visible region (>400 nm), which is ascribed to the Mie scattering induced by colloids [45]. Furthermore, the NPs solution showed intense cyan fluorescence owing to the AIE property of **M** under UV irradiation (Figure 1d, inset). On the contrary, the chloroform solution of **M** showed no emission. At the same time, the absolute fluorescence quantum yield of the **M** NPs was measured to be 22.29% (Figure S4). The fluorescence spectra of these samples further confirmed the good AIE behavior of the **M** NPs in water (Figure 1d). In this condition, the **M** dimers were incorporated in the confined space of **CTAB** micelles, leading to an intense restriction in molecular motion and resulting in strong emission. These preliminary studies suggested that the supramolecular dimer of **M** was successfully encapsulated into the NPs of **CTAB**. Notably, the obtained NPs can be stored for 1–2 weeks without any disruption and fluorescence bleaching.

**Figure 2:**
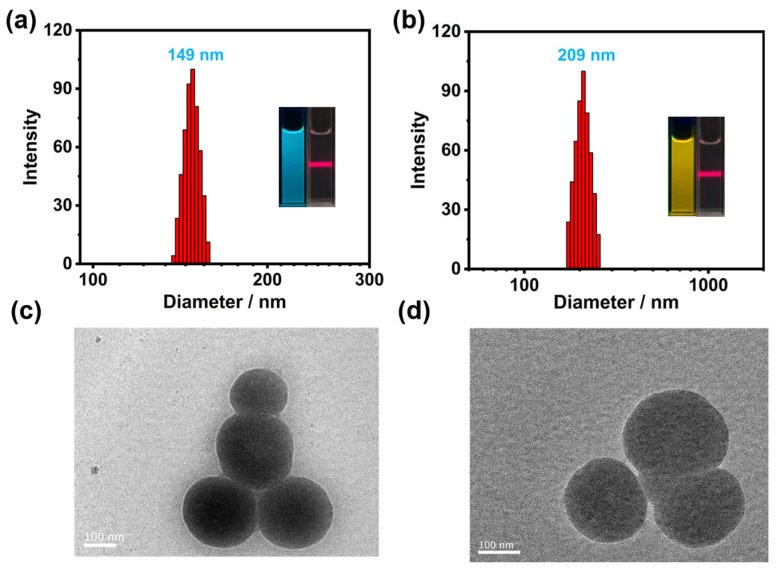
DLS data of (**a**) **M** NPs, inset: Photographs **M** NPs (left) under 365 nm UV lamp and the Tyndall effect of **M** NPs (right) and (**b**) **M**-**DBT** NPs, Photographs **M**-**DBT** NPs (left) under 365 nm UV lamp and the Tyndall effect of **M**-**DBT** NPs (right). TEM images of (**c**) **M** NPs and (**d**) **M**-**DBT** NPs. [**M**] = 5 × 10^−5^ M, [**DBT**] = 5 × 10^−7^ M, respectively.

The size and morphology of the obtained **M** NPs were further studied by transmission electron microscopy (TEM) and dynamic light scattering (DLS). DLS profile shows that the NPs are well-ordered nano-assemblies with a narrow distribution in size, indicative of an average hydro-dynamic diameter of 149 nm (Figure 2a). As shown in Figure 2c, the TEM image of **M** NPs indicated a spherical morphology with diameters ranging from 150 to 200 nm. Considering the good AIE behavior and stability of **M** in **CTAB** micelles, we further used them as a nanocarrier to accommodate acceptor chromophore to construct an artificial LHS in aqueous media.

**Figure 3:**
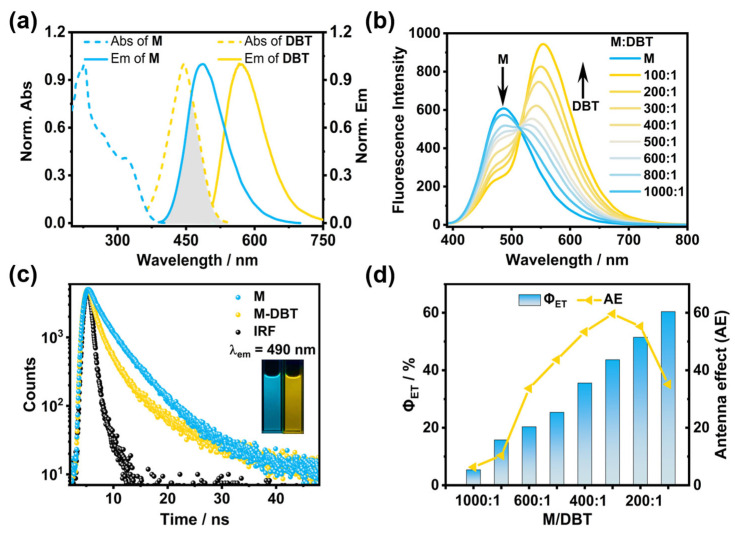
(**a**) Normalized absorption spectra of **M** and **DBT** (dashed lines) and their normalized fluorescence spectra (solid lines). (**b**) Fluorescence spectra of **M** dimer NPs with different concentrations of **DBT**. (**c**) Fluorescence decay profiles of NPs of **M** and **M-DBT**, λ_ex_ = 330 nm; inset: photographs of **M** and **M-DBT** under UV light. [**M**] = 5 × 10^−5^ M, [**DBT**] = 5 × 10^−7^ M. (**d**) Φ_ET_ and AE at different **M/DBT** ratios.

To enable efficient FRET, several prerequisites are needed: (1) the absorption of the acceptor should have a good overlap with the donor’s emission; (2) the donor and acceptor chromophores must be arranged in an appropriate manner; (3) the donor and acceptor must be sufficient close to each other. To meet these requirements, a commercially available dye **DBT** was selected as energy acceptor (Figure 3a). Because of the hydrophobic environment inside the NPs, **DBT** could be easily co-assembled into the NPs through mini-emulsifying **M** and **DBT** simultaneously. As the **DBT** molecules were evenly distributed inside the NPs, aggregation-caused quenching of **DBT** itself was avoided, enabling it to absorb excitation energy from **M**. The size and morphology of the **DBT**-leaded NPs were also characterized. The DLS measurements showed a narrow distribution with an average hydrodynamic diameter of 209 nm (Figure 2b). The TEM image showed that the diameter of the loaded NPs was approximately 200 nm, with a regular spherical morphology (Figure 2d).

With a titration of **DBT** to **M** NPs (Figure 3b), the fluorescence of the **M** dimer at 490 nm gradually decreased, while the emission peak of **DBT** at 555 nm increased significantly (λ_ex_ = 330 nm). Notably, the absolute quantum yield increased significantly from 22.29% to 44.61% with the addition of **DBT** (Figure S4b), implying that the **DBT** acceptor can harvest and release energy efficiently. A distinct change in the fluorescence color from cyan to bright yellow was observed (Figure 3c, inset), intuitively suggesting that FRET was realized from donor (**M**) to acceptor (**DBT**) in this system. The measurement of the fluorescence lifetime of the donor is another way to obtain insights into the energy transfer process. Therefore, fluorescence decay experiments were performed to verify the occurrence of the light-harvesting process. Both fluorescence lifetimes of **M** and **M**-**DBT** NPs were fitted as a double exponential decay curve (Figure 3c). The τ_1_ and τ_2_ of **M** dimer NPs monitored at 490 nm were determined to be 3.88 ns and 9.92 ns, respectively (Table S1). However, in the presence of **DBT** (**M**/**DBT** = 100/1), τ_1_ and τ_2_ were reduced to 2.26 ns and 8.91 ns, indicating that the excitation energy of **M** dimer was indeed transferred to **DBT**.

The antenna effect (AE) and energy-transfer efficiency (Φ_ET_) were further used to evaluate the light harvesting capability of the system. Herein, Φ_ET_ is the fluorescence quenching ratio of the donor at 555 nm, while AE is the amplification ratio of the **DBT** emission by exciting **M** and directly exciting **DBT**. As shown in Figure 3d, Φ_ET_ increased with the increase in **DBT** content. When the molar ratio of **M**/**DBT** = 100/1, it reached 60.4% (Table S3). A supramolecular network-based LHS formed by a similar UPy-derived TPE compound was reported previously. However, its Φ_ET_ was 37.2%, which might be due to the poor solubility of the compound [46]. It is noteworthy that AE of the **M**-**DBT** NPs reached the highest value of 59.61 when **M**/**DBT** = 300/1 (Table S4). In our previously reported covalent dimer systems, precursors containing only one AIE group as a donor were confirmed to be unworkable. In this work, we also employed **TPE** itself as a control donor to construct an LHS (Scheme S2). By contrast, a partial energy transfer and no antenna effect could be observed in the control system (Figure S7b). At the same time, the fluorescence color did not change, but the intensity decreased. These observations suggest that the formation of non-covalent dimers in the NPs is necessary for the light harvesting process.

**Figure 4:**
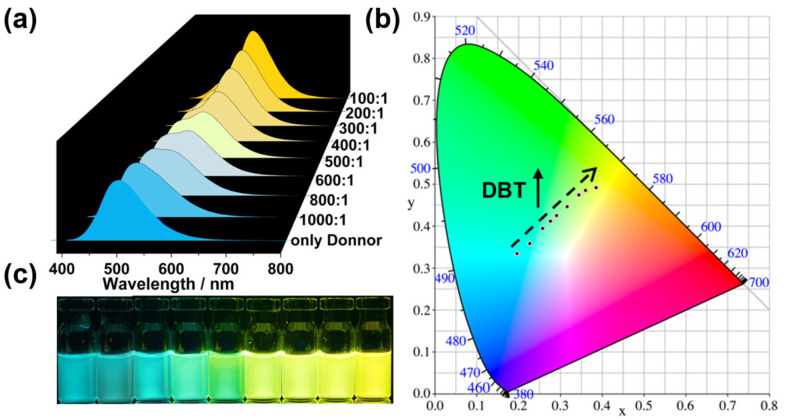
(**a**) The fluorescence spectra of **M** in water with different concentrations of **DBT**. (**b**) The CIE chromaticity diagram of photoluminescence color changes by varying the ratios of the chromophores. (**c**) Photographs of NPs in water with different **M/DBT** ratios. [**M**] = 5 × 10^−5^ M.

Owing to the flexibility of the construction process of the NPs, the system shows tunable emission behavior. Since it is not easy to know the exact color changes upon the addition of the acceptor by fluorescence spectra (Figure 4a), the Commission Internationale de l’Eclairage (CIE) chromaticity coordinates were used to intuitively show the change trend of the color. As shown in the CIE 1931 chromaticity diagram, the color of **M** NPs was located in the cyan region (Figure 4b). As the ratio of **DBT** increased from 1000:1 to 100:1, the fluorescence color of the LHS gradually changed from cyan to bright yellow, indicating an efficient energy transfer process. The emission photo at different **M**/**DBT** ratios is also exhibited in Figure 4c.

## 3. Materials and Methods

### 3.1. Materials

All chemicals were commercially available and used, unless otherwise stated, without further purification. If needed, solvents were dried by the literature procedures. All yields were given as isolated yields.

### 3.2. Methods

The ^1^H NMR spectra were recorded with a Bruker AVANCE III (300 MHz) spectrometer (Bruker Corporation, Billerica, MA, USA) and calibrated against the residual proton signal or natural abundance carbon resonance of the used deuterated solvent from tetramethylsilane (TMS) as the internal standard. The chemical shifts δ are indicated in ppm and the coupling constants *J* in Hz. The multiplicities are given as s (singlet), d (doublet), t (triplet), m (multiplet) and br (broad). High-resolution electrospray ionization mass spectra (HR-ESI-MS) were recorded on an Agilent Technologies 6540 UHD Accurate-Mass (Santa Clara, CA, USA). TEM characterizations were carried out on a JEM-2100 instrument (JEOL, Ltd. Tokyo, Japan). DLS was measured on a Zetasizer Nano ZS ZEN3600 (Malvern Panalytical Ltd., Malvern, UK). The solutions were filtrated, prior to use, through a filter (pore size: 0.45 μm). The UV-Vis absorption spectra were measured on a Perkin Elmer Lambda 35 UV-Vis Spectrometer (Waltham, MA, USA). Fluorescence measurements were performed on an Agilent Cary Eclipse spectrofluorometer (Agilent Technologies, Santa Clara, CA, USA). The fluorescence lifetimes were measured employing time-correlated single photon counting on a FLS980 instrument (Agilent Technologies, Santa Clara, CA, USA) with a pulsed xenon lamp. Analyses of fluorescence decay curves were subjected to fit a bi-exponential decay. The instrument response function (IRF) measures the scattering of laser excitation from non-fluorescent control samples to determine the fastest possible response of the detectors. The quantum yields were carried out on a FLS980 instrument with the integrating sphere.

### 3.3. Synthesis of M

**Scheme 2:**
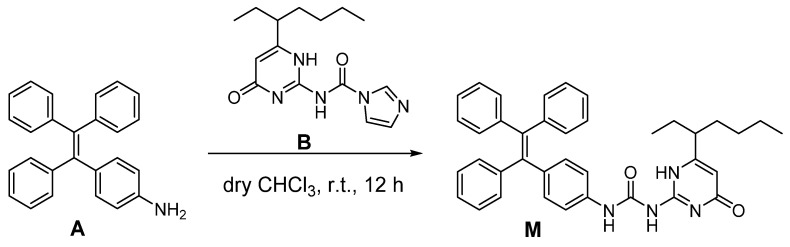
Synthesis of **M**. **A** stands for amino-substituted tetraphenylethylene, while **B** stands for the precursor of UPy.

The synthetic route of **M** is shown in Scheme 2. Compound **A** (1.0 g, 2.9 mmol) and compound **B** (1.31 g, 4.3 mmol) were dissolved in dry CHCl_3_ (20 mL) and stirred for 12 h under nitrogen at room temperature. After the completion of the reaction, cooling to room temperature, the organic layer was washed with 1 M HCl (40 mL × 2), saturated NaHCO_3_ (40 mL × 3), saturated NaCl (40 mL × 3), dried with anhydrous Na_2_SO_4_ and concentrated under reduced pressure. Column chromatography was performed using methanol in dichloromethane as an eluent to obtain the pure product as yellow solid (1.15 g, 68%). ^1^H NMR (300 MHz, CDCl_3_): δ (ppm) = 13.10 (s, 1H, N*H*), 12.12 (s, 2H, N*H*), 7.47 (d, *J* = 6 Hz, 2H, Ar*H*), 7.09–6.98 (m, 17H, Ar*H*), 5.90 (s, 1H, alkene-*H*), 2.33 (s, 1H, CH_3_CH_2_C*H*CH_2_-), 1.74–1.51 (m, 4H, alkyl-*H*), 1.35–1.20 (m, 4H, alkyl-*H*), 0.92–0.82 (m, 6H, -CH_2_C*H*_3_). ^13^C NMR (75 MHz, CDCl_3_): δ (ppm) = 173.0, 155.8, 154.8, 143.8, 140.6, 139.4, 136.4, 132.0, 131.4, 127.6, 126.4, 119.7, 106.6, 45.5, 33.0, 29.4, 26.7, 22.5, 13.9, 11.7. HR-ESI-MS: m/z [M + H]^+^ calcd for [C_38_H_39_N_4_O_2_]^+^ = 583.3068, found 583.3051.

### 3.4. Preparation of NPs

A solution of **M** in CHCl_3_ (50 mL, [**M**] = 10 mM) was dropped into an aqueous solution of **CTAB** (10 mL, [**CTAB**] = 1 mM). The obtained mixture was ultrasonicated for 0.5 h to produce the dispersed NPs.

## 4. Conclusions

In conclusion, an artificial light harvesting system with tunable fluorescence emission based on a supramolecular dimer was fabricated in an aqueous media. With the help of quadruple hydrogen bonds from the UPy group, the monomer **M** could form a non-covalent dimer and, subsequently, self-assemble into NPs by mini-emulsifying them with **CTAB**. By loading a hydrophobic dye **DBT** into the NPs, a light harvesting system based on two fluorophores (**M** and **DBT**) could be successfully constructed. Owing to the dynamic property of supramolecular self-assembly, the ratio of the donor and acceptor could be easily controlled and tunable emission was achieved. Compared to our previous works on LHSs, the preparation process in this study is much easier and more convenient. Such an efficient light harvesting system based on the non-covalent dimer may provide a promising platform for mimicking natural photosynthesis and shows great potential in dynamic fluorescent materials.

## Data Availability

Not applicable.

## Supplementary Materials

The following supporting information can be downloaded at: https://www.mdpi.com/article/10.3390/molecules27248876/s1, Figure S1: ^1^H NMR spectrum (300 MHz, CDCl_3_, 298 K) of **M**. Figure S2: ^13^C NMR spectrum (75 MHz, CDCl_3_, 298 K) of **M**. Figure S3: HR-ESI-MS spectrum of **M**. Figure S4: Absolute fluorescence quantum yields (Φ_f(abs)_) of (a) NPs of **M**, (b) NPs of **M-DBT** (**M**: **DBT** = 100: 1) upon excitation at 330 nm in aqueous solution, [**M**] = 5 × 10^−5^ M, [**DBT**] = 5 × 10^−7^ M, respectively. Figure S5: Fluorescence spectrum of NPs of **M** and NPs of **M-DBT** in aqueous solution. [**M**] = 5 × 10^−5^ M, [**DBT**] = 5 × 10^−7^ M, respectively. Figure S6: Fluorescence spectrum of **M-DBT** (yellow line) when excited at 330 nm and **M-DBT** (black line) excited at 490 nm. The cyan line represents the fluorescence spectrum of **M**, which was normalized according to the fluorescence intensity of yellow line at 490 nm. [**M**] = 5 × 10^−5^ M, [**DBT**] = 5 × 10^−7^ M, respectively. Figure S7: (a) Fluorescence spectra of **M** and **M-DBT** upon excitation at 330 nm. (b) Fluorescence spectra of compound **TPE** and **TPE**-**DBT** upon excitation at 330 nm. All these compounds exist as NPs in water. [**M**] = 5 × 10^−5^ M, [**TPE**] = 5 × 10^−5^ M, respectively.; Table S1: title Fluorescence lifetimes of **M** NPs and **M-DBT** NPs monitored at 490 nm upon excitation at 365 nm in aqueous solution. [**M**] = 5 × 10^−5^ M, [**DBT**] = 5 × 10^−7^ M, respectively. Table S2: Fluorescence quantum yields (**Φ**_f(abs)_) of (a) **M** NPs, (b) **M-DBT** NPs. [**M**] = 5 × 10^−5^ M, [**DBT**] = 5 × 10^−7^ M, respectively. Table S3: Energy-transfer efficiency with different **M**/**DBT** ratio. Table S4: Antenna effect with different **M**/**DBT** ratio.; Scheme S1: Synthesis of **M**. Scheme S2: Chemical structure of **TPE**. References [47,48,49,50] are cited in the supplementary materials.

## Sample Availability

Samples of the compounds are not available from the authors.
