# Supplementary material for: Non-Covalent Dimer as Donor Chromophore for Constructing Artificial Light-Harvesting System in Water
Source: Molecules. 2022 Dec 14;27(24):8876. doi: 10.3390/molecules27248876 (PMC9781999; doi:10.3390/molecules27248876)
Supplement: Supplementary file 1 [file molecules-27-08876-s001.zip › molecules-2072571-supplementary.pdf]

Non-Covalent Dimer as Donor Chromophore for Constructing Artificial Light-Harvesting System in Water

Liangliang Zhang 1, Hongwei Qian 1, Zhiying Wu 1, Qiaona Zhang 1, Shengke Li 2,*, Ming Cheng 3,* and Tangxin Xiao 1,*

1 Jiangsu Key Laboratory of Advanced Catalytic Materials and Technology,

School of Petrochemical Engineering, Changzhou University, Changzhou 213164, China;

2 State Key Laboratory of Quality Research in Chinese Medicine, Institute of Chinese Medical Sciences, University of Macau, Macau 999078, China

3 Jiangsu Key Laboratory of Advanced Organic Materials, School of Chemistry and Chemical Engineering, Nanjing University, 163 Xianlin Avenue, Nanjing 210023, China

** Correspondence: lisk@njust.edu.cn (S.L.); ming.cheng2015@foxmail.com (M.C.);*

xiaotangxin@cczu.edu.cn (T.X.)

Table of contents

1. Materials, methods, and abbreviations……………………………….S2
2. Synthesis of compound **M**…………………………………………..S4
3. Fluorescence lifetime measurements quantum yields………..……..S6
4. Energy-transfer efficiency calculation……………………………….S7
5. Antenna effect calculation……………….……………….………….S9
6. Control experiment of **TPE-DBT** for light-harvesting…………….……S10
7. References……………………….………………………..…….…....S11

1. Materials, methods, and abbreviations

General

All chemicals, reagents and solvents were purchased from commercial suppliers and used, unless otherwise stated, without further purification. If needed, solvents were dried by literature known procedures. All yields were given as isolated yields. Compound **A**[S1] and compound **B**[S2] were prepared according to literature procedure.

NMR spectroscopy

The ^{1}H NMR and ^{13}C NMR spectra were recorded with a Bruker AVANCE III (300 MHz) spectrometer and calibrated against the residual proton signal or natural abundance carbon resonance of the used deuterated solvent from tetramethylsilane (TMS) as the internal standard. The chemical shifts δ are indicated in ppm and the coupling constants J in Hz. The multiplicities are given as s (singlet), d (doublet), dd (doublet of doublets), t (triplet), and m (multiplet).

Mass spectrometry

High-resolution electrospray ionization mass spectra (HR-ESI-MS) were recorded on an Agilent Technologies 6540 UHD Accurate-Mass. High performance liquid chromatography (HPLC) analysis was performed on Agilent 1260 HPLC.

Transmission electron microscope (TEM)

TEM investigations were carried out on a JEM-2100 instrument.

Dynamic light scattering (DLS)

DLS measurements were carried out on a Brookhaven BI-9000AT system, equipped with a 200 mW polarized laser source (λ = 514 nm) at a scattering angle of 90°. All samples were prepared according to the corresponding procedures mentioned above.

UV-Vis spectroscopy

The UV-Vis absorption spectra were measured on a Perkin Elmer Lambda 35 UV-Vis Spectrometer.

Fluorescence spectroscopy

Fluorescence measurements were performed on an Agilent Cary Eclipse spectrofluorometer.

Fluorescence lifetimes

The fluorescence lifetimes were measured employing time correlated single photon counting on a FLS980 instrument with a pulsed xenon lamp. Analysis of fluorescence decay curves were subjected to fit a mono-exponential or bi-exponential decay. The instrument response function (IRF) measures the scattering of laser excitation from non-fluorescent control samples to determine the fastest possible response of the detectors.

Quantum yields

The quantum yields were carried out on a FLS980 instrument with the integrating sphere.

CIE coordinates

The CIE (Commission Internationale de l'Eclairage) 1931 coordinates were calculated with the method of color matching functions.

Abbreviations

NPs = nanoparticles; DCM = dichloromethane; M = mol/L

2. Synthesis of compound M

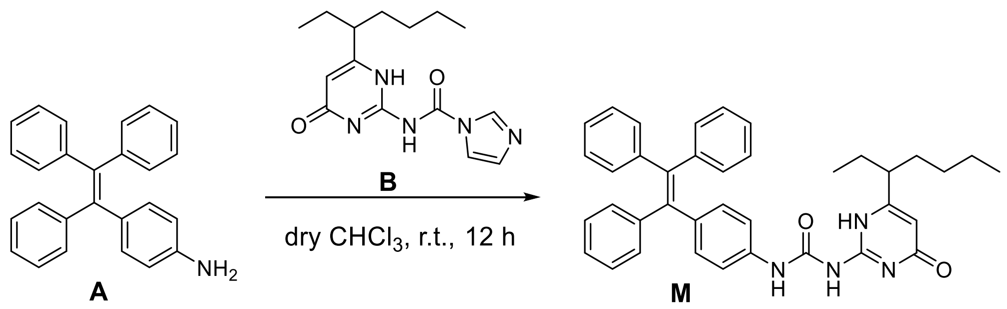

Scheme S1. Synthesis of **M**.

Synthesis of M

Compound **A** (1.0 g, 2.9 mmol) and compound **B** (1.31 g, 4.3 mmol) were dissolved in dry $CHCl_3$ (20 mL) and stirred for 12 h under nitrogen at room temperature. After the completion of the reaction, cooling to room temperature, the organic layer was washed with 1 M HCl (40 mL × 2), saturated $NaHCO_3$ (40 mL × 3), saturated NaCl (40 mL × 3), dried with anhydrous Na_2SO_4 and concentrated under reduced pressure. Column chromatography was performed using methanol in dichloromethane as an eluent to get the pure product as yellow solid (1.15 g, 68%). ^{1}H NMR (300 MHz, $CDCl_3$): δ (ppm) = 13.10 (s, 1H, N*H*), 12.12 (s, 2H, N*H*), 7.47 (d, *J* = 6 Hz, 2H, Ar*H*), 7.09-6.98 (m, 17H, Ar*H*), 5.90 (s, 1H, alkene-*H*), 2.33 (s, 1H, $CH_3CH_2C\mathit{H}CH_2$-), 1.74-1.51 (m, 4H, alkyl-*H*), 1.35-1.20 (m, 4H, alkyl-*H*), 0.92-0.82 (m, 6H, -$CH_2C\mathit{H}_3$). ^{13}C NMR (75 MHz, $CDCl_3$): δ (ppm) = 173.0, 155.8, 154.8, 143.8, 140.6, 139.4, 136.4, 132.0, 131.4, 127.6, 126.4, 119.7, 106.6, 45.5, 33.0, 29.4, 26.7, 22.5, 13.9, 11.7. HR-ESI-MS: m/z $[M + H]^+$ calcd for $[C_{38}H_{39}N_4O_2]^+$ = 583.3068, found 583.3051.

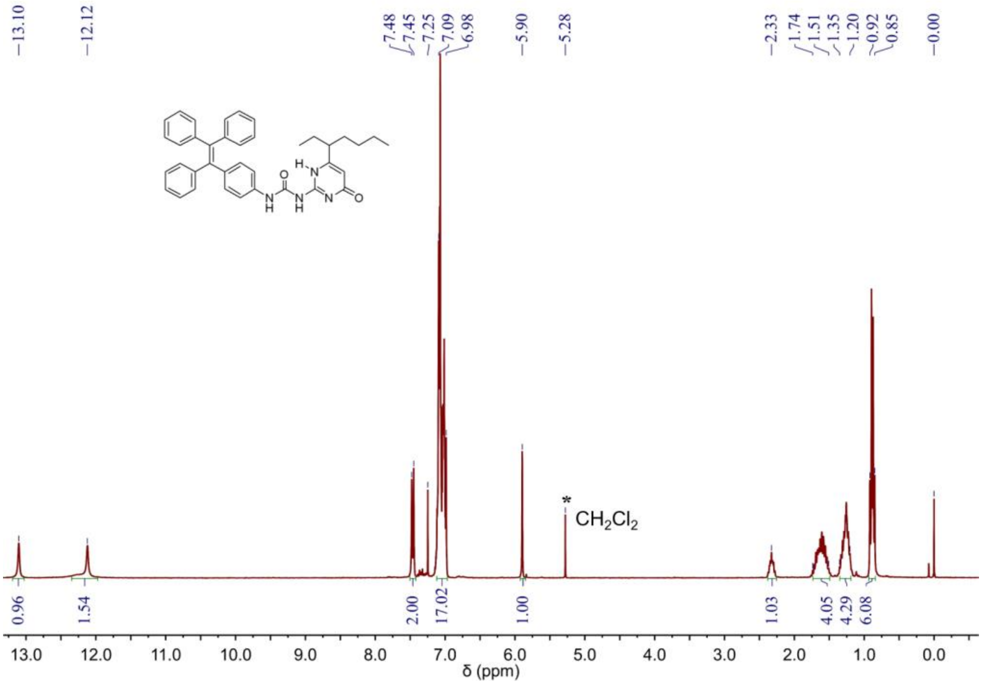

Figure S1. ^{1}H NMR spectrum (300 MHz, $CDCl_3$, 298 K) of **M**.

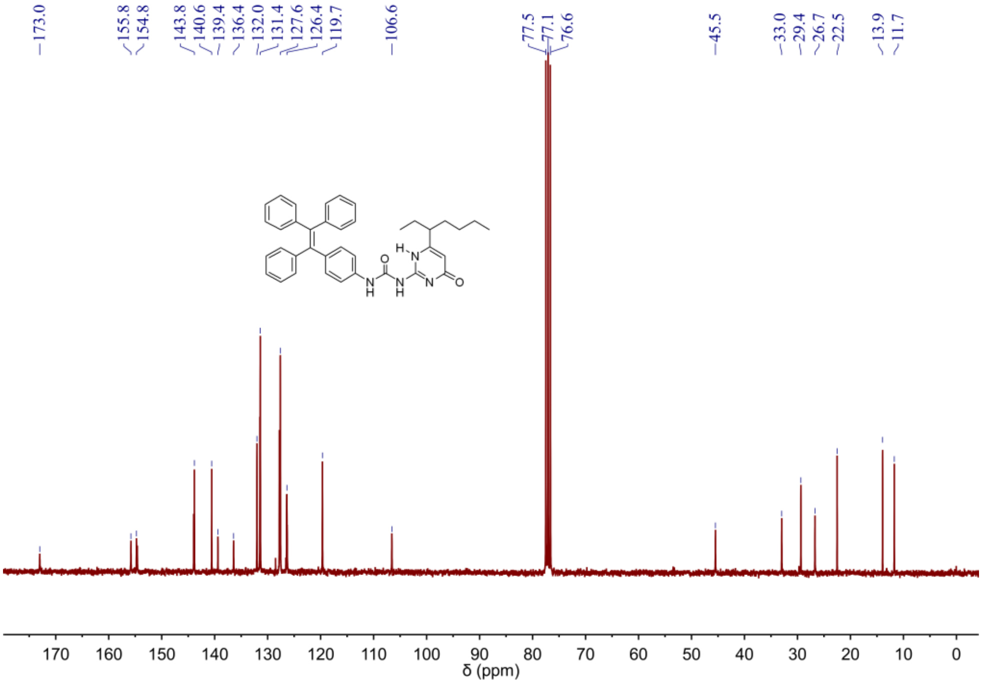

Figure S2. ^{13}C NMR spectrum (75 MHz, $CDCl_3$, 298 K) of **M**.

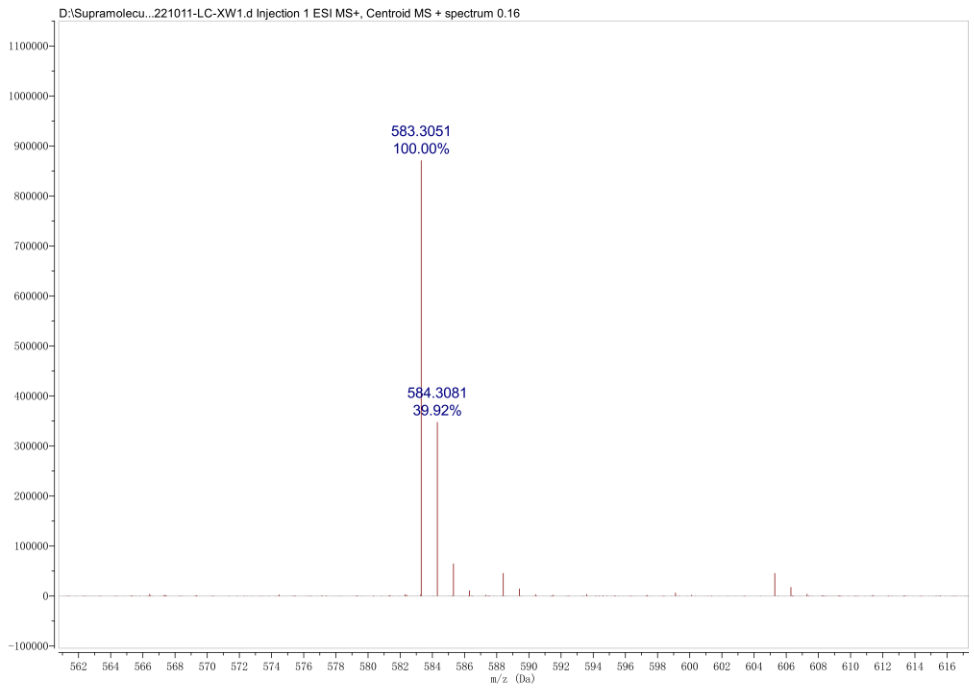

Figure S3. HR-ESI-MS spectrum of **M**.

3. Fluorescence lifetimes and quantum yields

Table S1. Fluorescence lifetimes of **M** NPs and **M-DBT** NPs monitored at 490 nm upon excitation at 365 nm in aqueous solution. [**M**] = 5 ×10^{-5} M, [**DBT**] = 5 ×10^{-7} M, respectively.

Sample	τ_1	RW1%	τ_2	RW2%	τ	χ^2
**M**	3.88	61.3%	9.92	38.7%	6.22	1.108
**M-DBT** (**M** : **DBT** = 100 : 1)	2.26	51.72%	8.91	48.28%	5.47	1.191

Table S2. Fluorescence quantum yields ($\Phi_{f(abs)}$) of (a) **M** NPs, (b) **M-DBT** NPs. [**M**] = 5 × 10^{-5} M, [**DBT**] = 5 × 10^{-7} M, respectively.

Sample	Fluorescence quantum yields ($\Phi_{f(abs)}$)
**M**	22.29%
**M-DBT** (**M** : **DBT** = 100 : 1)	44.61%

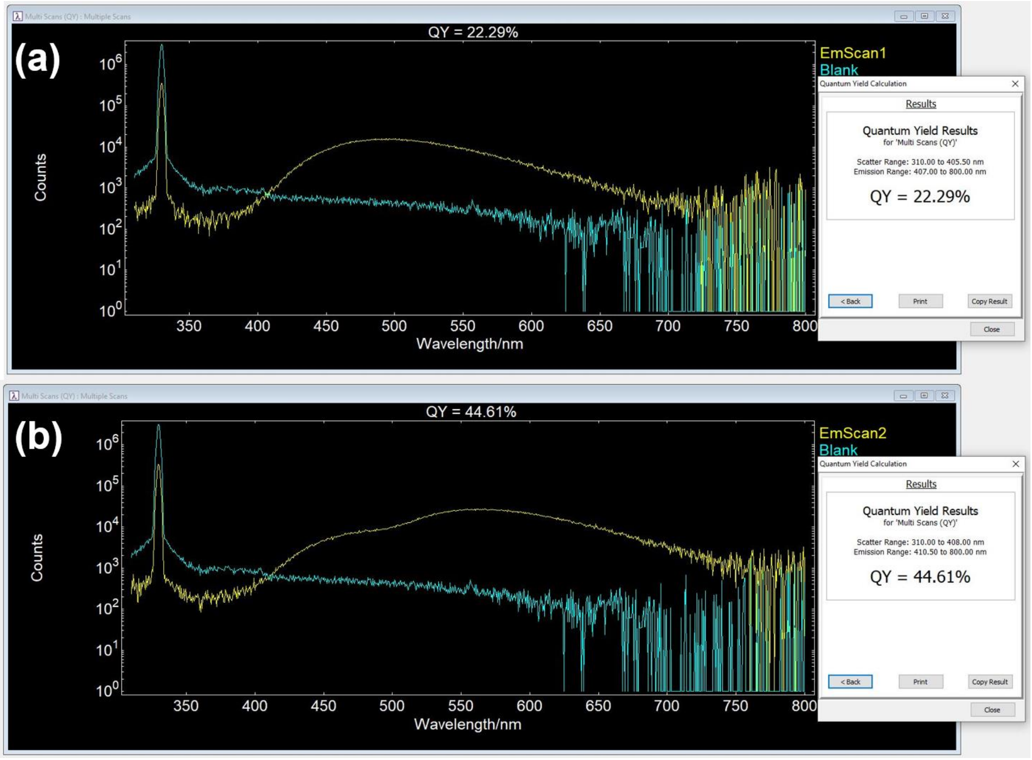

Figure S4. Absolute fluorescence quantum yields ($\Phi_{f(abs)}$) of (a) NPs of **M**, (b) NPs of **M-DBT** (**M** : **DBT** = 100 : 1) upon excitation at 330 nm in aqueous solution, [**M**] = 5 × 10^{-5} M, [**DBT**] = 5 × 10^{-7} M, respectively.

4. Energy-transfer efficiency calculation

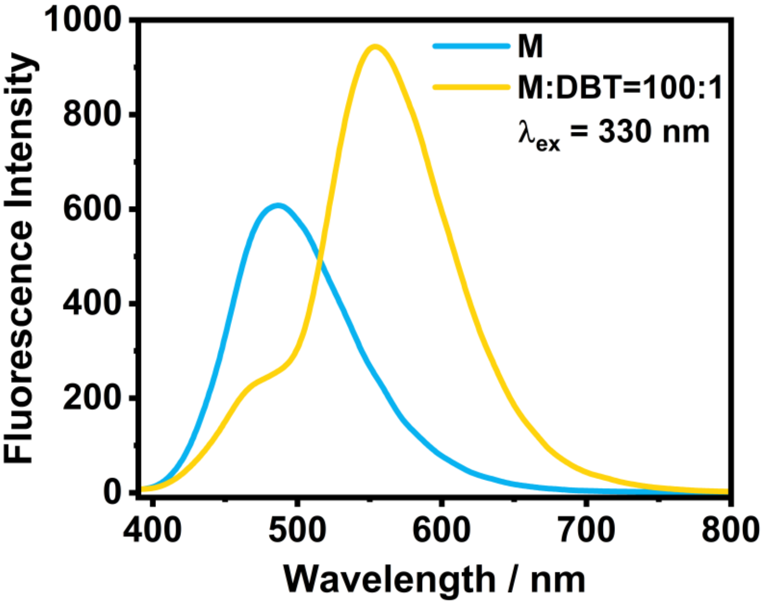

Figure S5. Fluorescence spectrum of NPs of **M** and NPs of **M-DBT** in aqueous solution. [**M**] = 5 ×10^{-5} M, [**DBT**] = 5 ×10^{-7} M, respectively.

Energy-transfer efficiency (Φ_{ET}) was calculated from fluorescence spectra by the equation S1[S3-S5]:

$$\mathbf{\Phi_{ET} = 1 - I_{DA} / I_D} \text{ (eq. S1)}$$

Where $\mathbf{I_{DA}}$ and $\mathbf{I_D}$ are the fluorescence intensities of the donor (**M**) at 490 nm (Fig. S5, cyan line) and the donor and acceptor (**M-DBT**) (Fig. S5, yellow line), respectively, when excited at 330 nm.

The $\mathbf{\Phi_{ET}}$ was calculated to be 60.4% under the condition of [**M**] = 5 × 10^{-5} M, [**DBT**] = 5 × 10^{-7} M, (λ_{ex} = 330 nm and λ_{em} = 490 nm).

Similarly, the $\mathbf{\Phi_{ET}}$ of other **M/DBT** ratios could also be calculated according to this equation.

Table S3. Energy-transfer efficiency with different **M/DBT** ratio.

**Sample**	**Concentration, respectively**	**Energy-transfer efficiency ($\mathbf{\Phi_{ET}}$)**
**M-DBT** (**M : DBT** = 100 : 1)	[**M**] = 5×10^{-5} M<br>[**DBT**] = 5×10^{-7} M	60.4%
**M-DBT** (**M** : **DBT** = 200 : 1)	[**M**] = 5×10^{-5} M<br>[**DBT**] = 2.5×10^{-7} M	51.5%
**M-DBT** (**M** : **DBT** = 300 : 1)	[**M**] = 5×10^{-5} M<br>[**DBT**] = 1.66×10^{-7} M	43.7%
**M-DBT** (**M** : **DBT** = 400 : 1)	[**M**] = 5×10^{-5} M<br>[**DBT**] = 1.25×10^{-7} M	35.6%
**M-DBT** (**M : DBT** = 500 : 1)	[**M**] = 5×10^{-5} M<br>[**DBT**] = 1.0×10^{-7} M	25.4%
**M-DBT** (**M : DBT** = 600 : 1)	[**M**] = 5×10^{-5} M<br>[**DBT**] = 8.3×10^{-8} M	20.4%
**M-DBT** (**M : DBT** = 800 : 1)	[**M**] = 5×10^{-5} M<br>[**DBT**] = 6.25×10^{-8} M	15.8%
**M-DBT** (**M : DBT** =1000 : 1)	[**M**] = 5×10^{-5} M<br>[**DBT**] = 5×10^{-8} M	5.4%

5. Antenna effect calculation

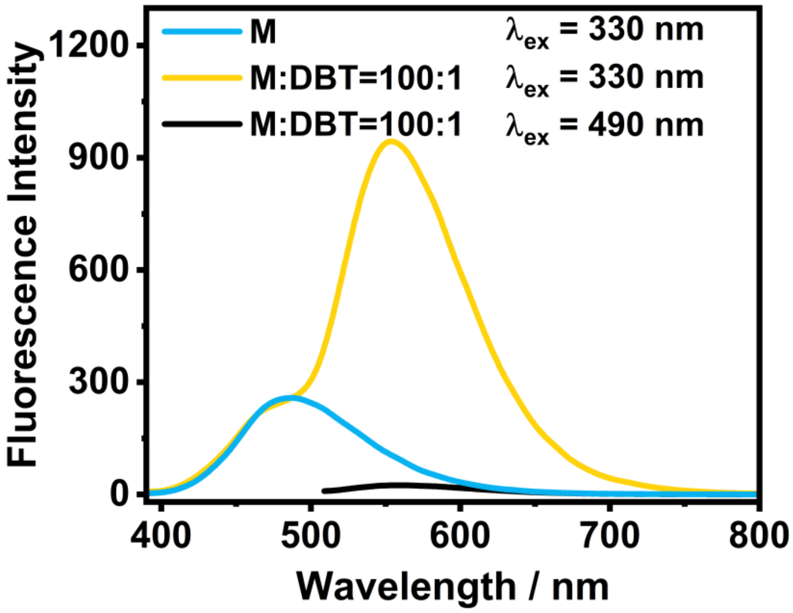

Figure S6. Fluorescence spectrum of **M-DBT** (yellow line) when excited at 330 nm and **M-DBT** (black line) excited at 490 nm. The cyan line represents the fluorescence spectrum of **M**, which was normalized according to the fluorescence intensity of yellow line at 490 nm. [**M**] = 5×10^{-5} M, [**DBT**] = 5×10^{-7} M, respectively.

The antenna effect (AE) was calculated based on the emission spectra according to equation S2[S3-S5]:

$$\mathbf{AE = (I_{DA,330} - I_{D,330}) / I_{DA,490}} \text{ (eq. S2)}$$

Where $\mathbf{I_{DA,330}}$ is the fluorescence intensity of **DBT** at 555 nm with the excitation of the donor at 330 nm (Fig. S6, yellow line). $\mathbf{I_{DA,490}}$ is the fluorescence intensity of **DBT** at 555 nm with the direct excitation of the acceptor at 490 nm (Fig. S6, black line). $\mathbf{I_{D,330}}$ is the normalized fluorescence intensity of individual **M** at 555 nm (Fig. S6, cyan line).

The AE value was calculated as 35.1 under the condition of [**M**] = 5×10^{-5} M, [**DBT**] = 5×10^{-7} M, respectively.

Similarly, the antenna effect of other **M/DBT** ratios was also calculated according to this equation.

Table S4. Antenna effect with different **M**/**DBT** ratio.

Sample	Concentration, respectively	Antenna effect (AE)
**M-DBT** (**M : DBT** = 100 : 1)	[**M**] = 5×10^{-5} M<br>[**DBT**] = 5×10^{-7} M	35.11
**M-DBT** (**M** : **DBT** = 200 : 1)	[**M**] = 5×10^{-5} M<br>[**DBT**] = 2.5×10^{-7} M	55.24
**M-DBT** (**M** : **DBT** = 300 : 1)	[**M**] = 5×10^{-5} M<br>[**DBT**] = 1.66×10^{-7} M	59.61
**M-DBT** (**M** : **DBT** = 400 : 1)	[**M**] = 5×10^{-5} M<br>[**DBT**] = 1.25×10^{-7} M	53.38
**M-DBT** (**M : DBT** = 500 : 1)	[**M**] = 5×10^{-5} M<br>[**DBT**] = 1.0×10^{-7} M	43.68
**M-DBT** (**M : DBT** = 600 : 1)	[**M**] = 5×10^{-5} M<br>[**DBT**] = 8.3×10^{-8} M	33.69
**M-DBT** (**M : DBT** = 800 : 1)	[**M**] = 5×10^{-5} M<br>[**DBT**] = 6.25×10^{-8} M	10.33
**M-DBT** (**M : DBT** =1000 : 1)	[**M**] = 5×10^{-5} M<br>[**DBT**] = 5×10^{-8} M	6.3

6. Control experiment of TPE-DBT for light-harvesting

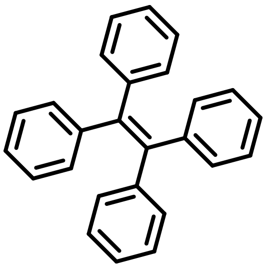

TPE

Scheme S2. Chemical structure of **TPE**.

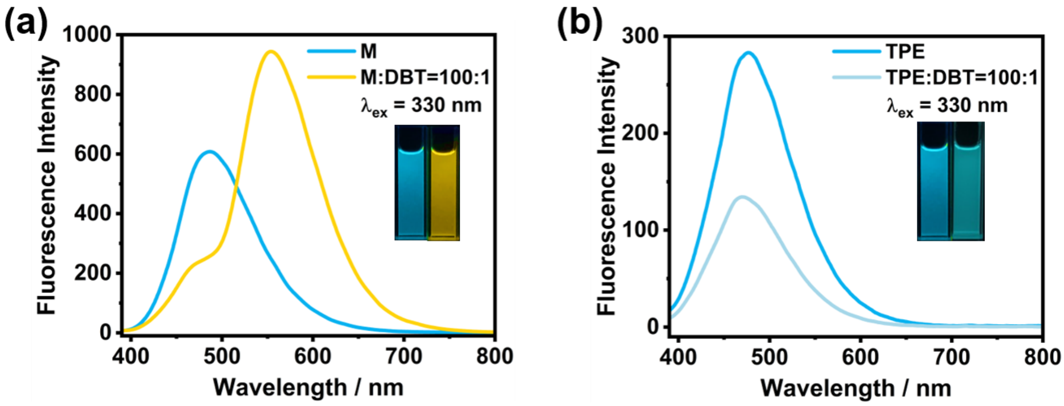

Figure S7. (a) Fluorescence spectra of **M** and **M-DBT** upon excitation at 330 nm. (b) Fluorescence spectra of compound **TPE** and **TPE-DBT** upon excitation at 330 nm. All these compounds are existed as NPs in water. [**M**] = 5 × 10^{-5} M, [**TPE**] = 5 × 10^{-5} M, respectively.

7. References

[S1] Li, W.-B.; Luo, W.-J.; Li, K.-X.; Yuan, W.-Z.; Zhang, Y.-M., Aggregation-induced phosphorescence and mechanochromic luminescence of a tetraphenylethene-based gold(I) isocyanide complex. *Chin. Chem. Lett.* **2017,** *28*, 1300-1305.

[S2] Keizer, H. M.; Sijbesma, R. P.; Meijer, E. W. Eur. *J. Org. Chem.* **2004**, *2004*, 2553-2555.

[S3] T. Xiao, H. Wu, G. Sun, K. Diao, X. Wei, Z. Y. Li, X. Q. Sun and L. Wang, An efficient artificial light-harvesting system with tunable emission in water constructed from a H-bonded AIE supramolecular polymer and Nile Red, *Chem. Commun.*, 2020, **56**, 12021-12024.

[S4] Li, X.; Yu, S.; Shen, Z.; Wang, R.; Zhang, W.; Nunez-Delgado, A.; Han, N.; Xing, L. B., Supramolecular assemblies working as both artificial light-harvesting system and nanoreactor for efficient organic dehalogenation in aqueous environment. *J. Colloid. Interface Sci.* **2022,** *617*, 118-128.

[S5] Liu, Z.; Sun, X.; Dai, X.; Li, J.; Li, P.; Liu, Y., Sulfonatocalix[4]arene-based light-harvesting amphiphilic supramolecular assemblies for sensing sulfites in cells. *J. Mater. Chem. C* **2021,** *9*, 1958-1965.
